# Ridle for sparse regression with mandatory covariates with application to the genetic assessment of histologic grades of breast cancer

**DOI:** 10.1186/s12874-017-0291-y

**Published:** 2017-01-25

**Authors:** Jing Zhai, Chiu-Hsieh Hsu, Z. John Daye

**Affiliations:** Epidemiology and Biostatistics Department, University of Arizona, Tucson, USA

**Keywords:** Gene expression analysis, Lasso, Linear models, Penalized regression, Ridge, Variable selection

## Abstract

**Background:**

Many questions in statistical genomics can be formulated in terms of variable selection of candidate biological factors for modeling a trait or quantity of interest. Often, in these applications, additional covariates describing clinical, demographical or experimental effects must be included a priori as mandatory covariates while allowing the selection of a large number of candidate or optional variables. As genomic studies routinely require mandatory covariates, it is of interest to propose principled methods of variable selection that can incorporate mandatory covariates.

**Methods:**

In this article, we propose the ridge-lasso hybrid estimator (ridle), a new penalized regression method that simultaneously estimates coefficients of mandatory covariates while allowing selection for others. The ridle provides a principled approach to mitigate effects of multicollinearity among the mandatory covariates and possible dependency between mandatory and optional variables. We provide detailed empirical and theoretical studies to evaluate our method. In addition, we develop an efficient algorithm for the ridle. Software, based on efficient Fortran code with R-language wrappers, is publicly and freely available at https://sites.google.com/site/zhongyindaye/software.

**Results:**

The ridle is useful when mandatory predictors are known to be significant due to prior knowledge or must be kept for additional analysis. Both theoretical and comprehensive simulation studies have shown that the ridle to be advantageous when mandatory covariates are correlated with the irrelevant optional predictors or are highly correlated among themselves. A microarray gene expression analysis of the histologic grades of breast cancer has identified 24 genes, in which 2 genes are selected only by the ridle among current methods and found to be associated with tumor grade.

**Conclusions:**

In this article, we proposed the ridle as a principled sparse regression method for the selection of optional variables while incorporating mandatory ones. Results suggest that the ridle is advantageous when mandatory covariates are correlated with the irrelevant optional predictors or are highly correlated among themselves.

**Electronic supplementary material:**

The online version of this article (doi:10.1186/s12874-017-0291-y) contains supplementary material, which is available to authorized users.

## Background

Many essential problems in statistical genomics may be formulated in terms of variable selection of candidate biological factors for modeling of some trait or quantity of interest [1–3]. Often, additional covariates describing clinical, demographical, or other experimental factors must be included a priori as mandatory covariates while allowing the selection of possibly a large number of candidate or optional variables. Substantial progress has been made recently in the analysis of high-dimensional data with sparse regression methods. The lasso was proposed that induces sparsity using an *L*1-norm penalty on all coefficients [4]. With the introduction of computationally efficient algorithms [5, 6], the lasso has since become a widely-applied variable selection method. Other methods for sparse regression include the smoothly clipped absolute deviation (SCAD) [7], adaptive lasso [8], Dantzig selector [9], etc. However, these methods were not designed for applications with mandatory covariates. An ad hoc approach is often employed where the response is regressed on mandatory covariates without penalization, as if in an ordinary least squares (OLS), while penalized regression is applied upon the optional variables, independently of the mandatory ones, to achieve variable selection. However, standard statistical principle advocates the consideration of all covariates simultaneously in order to account for complex dependencies among covariates. By penalizing coefficients disparately on some of the variables while not on others, this approach can yield both poor prediction accuracy and unreliable selection of optional variables. As mandatory covariates are routinely encountered in genomic-data analysis, it is of interest to develop a principled approach towards sparse regression with mandatory covariates. In this article, we consider the problem of efficient estimation of coefficients of mandatory covariates and simultaneous variable selection of optional variables.

Cancer arises as a disorder of the cell life cycle that leads to excessive cell proliferation and poor differentiation. Pathologists often use grading systems to measure the degree of cell differentiation in tumors [10, 11]. Tumor grade is one of the most important indicators for clinicians to guide treatment options and make prognosis for patients [12]. Histologic grade of breast cancer is representative of its aggressive potential [13]. Cancer cells with higher grades tend to be more aggressive and require quite different treatment strategies than those with lower grades. Due to the importance of tumor grade as an essential measure in clinical prognosis, treatment and of the survival of breast cancer patients, understanding genetic factors that may be predictive of tumor grade has become a desideratum of current research in breast cancers. In this article, we will propose a principled method to identify genes which may affect tumor grade while accounting for their clinical phenotypes such as age at diagnosis, p53 sequence mutation status, etc. by incorporating them as mandatory covariates.

We propose the *rid*ge-*l*asso hybrid *e*stimator (ridle), a novel penalized regression procedure that can simultaneously estimate coefficients of mandatory covariates while allowing selection for others. The ridle employs the *L*2-norm penalty to estimate mandatory coefficients and the *L*1-norm penalty to perform variable selection on the optional set. The *L*2-norm penalty has been successfully employed in ridge regression to efficiently estimate coefficients under a spectrum of dependency structures [14–16]. In this article, we provide theoretical, simulation, and real-data analysis to suggest the ridle as an efficient method for sparse regression with mandatory covariates. In particular, we will show that the ridle can achieve improved prediction accuracy and variable selection under commonly encountered scenarios when (1) the mandatory covariates are highly correlated among themselves or (2) the mandatory variables are correlated with the optional ones.

The rest of the article is presented as follows: “Methods” section introduces the ridle procedure, where an efficient algorithm is introduced and theoretical results are provided to suggest the efficacy of the ridle for sparse regression under mandatory predictors. “Results” section evaluates our method on simulated data. Further, we apply our method to a gene selection analysis of microarray data, where we identified more genes in breast-cancer related pathways with the ridle. Additionally, the ridle is the only method that identified two genes AREG and TRPM4 from the ErbB signaling pathway and ion-channel family, respectively, which are known to be related to cancer. Further discussions are provided in “Discussion” section, and we conclude with “Conclusions” section.

## Methods

### The Ridle

Consider the linear regression model,

$$\mathbf{y} = \mathbf{X} {\beta}^{0} + \boldsymbol{\epsilon}, $$

where **y** is an n-dimensional vector of random responses, **X**=(**x**_1_,**x**_2_,…,**x**_*d*_) is the *n*×*d* design matrix, ${\beta }^{0} = (\beta _{1}^{0}, \beta _{2}^{0}, \ldots, \beta _{d}^{0})$ is a vector of regression parameters, and ***ε*** is an *n*-dimensional vector of independent and identically distributed (i.i.d.) random variables with mean 0 and variance *σ* ^2^. We further assume that the response is centered and each predictor **x**_*j*_=(*x*_1*j*_,*x*_2*j*_,…,*x*_*nj*_) is standardized to have variance 1.

We define the *ridle* as,

1 $$  \hat{{\beta}} (\lambda_{1},\lambda_{2}) = \arg \min_{{\beta}} \, \Big\{||\mathbf{y} - \mathbf{X}{\beta}||^{2} + \lambda_{1} \sum_{j\in \mathcal{O}} |\beta_{j}| + \lambda_{2} \sum_{j\in \mathcal{M}} \beta_{j}^{2} \Big\},  $$

where $\mathcal {O}$ and $\mathcal {M}$ are non-intersecting subsets of the indices $\mathcal {I}=\{ 1,2,\ldots,d \}$ such that $\mathcal {O} \cup \mathcal {M} = \mathcal {I}$. Subsets $\mathcal {O}$ and $\mathcal {M}$ comprise, respectively, indices of optional and mandatory variables. The ridle penalizes coefficients in $\mathcal {O}$ by the *L*1-norm penalty and coefficients in $\mathcal {M}$ by the *L*2-norm penalty. It allows variable selection, as in the lasso, for predictors in $\mathcal {O}$ and estimation without selection, as in the ridge, for predictors in $\mathcal {M}$. If *λ*_2_ is equal to 0, the ridle is equivalent to thresholding the coefficients of some predictors for variable selection and estimating the rest without penalization. As the lasso penalty is applied to optional variables while no penalization is imposed on coefficients in $\mathcal {M}$, we call the special case of the ridle when *λ*_2_=0 as the $\mathcal {M}$-unpenalized lasso.

For further insight, we examine the ridle estimator under two special situations.

### Ridle estimator in two special cases

#### Orthogonal design case.

For **X** ^*T*^ **X**/*n* equal to the identity matrix **I**, we can easily obtain the ridle solution in terms of the ordinary least squares estimates $\hat {{\beta }}_{j}(\text {ols})$,

2 $$ \hat{{\beta}}_{j} = \left\{ \begin{array}{ll} \text{sign} (\hat{{\beta}}_{j}(\text{ols})) (|\hat{{\beta}}_{j}(\text{ols})| - \frac{\lambda_{1}}{2n})^{+}, & j\in \mathcal{O} \\ (1+\frac{\lambda_{2}}{n})^{-1}\hat{{\beta}}_{j}(\text{ols}), & j\in \mathcal{M} \\ \end{array} \right.  $$

where (·)^+^ denotes the positive part of the value, such that the expression is set to 0 for negative quantities. The ridle estimates equate to those of the lasso for $j \in \mathcal {O}$ and the ridge for $j \in \mathcal {M}$. When *λ*_2_=0, the $\mathcal {M}$-unpenalized lasso estimates equate to those of the lasso for $j \in \mathcal {O}$ and the OLS for $j \in \mathcal {M}$. It is clear that, when the design matrix is orthogonal, the *L*1-norm and *L*2-norm penalties work independently to penalize coefficients with indices in $\mathcal {O}$ and $\mathcal {M}$, respectively. The situation is more involved when predictors are correlated.

#### Two-predictor case

**Fig. 1:**
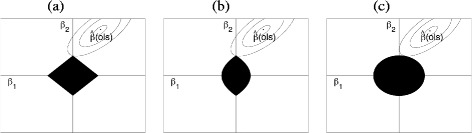
Penalty contour when *d*=2 for (**a**) lasso, (**b**) ridle, and (**c**) ridge regressions

Consider the case when *d*=2. Let $\mathcal {O}=\{1\}$ and $\mathcal {M}=\{2\}$ for the ridle estimates. Figure 1 presents the penalty contours of the lasso, ridle, and ridge estimators. The ellipses centered at the OLS solutions are the contours of the quadratic loss function,

$$({\beta}-\hat{{\beta}}(\text{ols}))^{T} \mathbf{X}^{T} \mathbf{X} ({\beta}-\hat{{\beta}}(\text{ols})), $$

plus a constant. With standardized predictors, these elliptical contours are at a ± 45°angle to the horizontal axes. Solutions occur when the ellipses first contact the penalty contours.

In Fig. 1 a, we obtain the lasso solution as an ellipse hits a corner of the lasso penalty contour, setting *β*_1_ to 0. In Fig. 1 c, we see that the ridge penalty contour is circular, and an ellipse hitting the penalty contour gives nonzero estimates. The ridle penalty is described in Fig. 1 b. It has both the characteristics of the lasso and ridge with an oval shape along the horizontal and sharp corners on the vertical axis. The ridle solution occurs when an ellipse centered on the OLS estimates hits a sharp corner on the vertical axis, yielding *β*_1_=0 and a nonzero *β*_2_. Thus, we see that the ridle may provide sparse solutions for coefficients in $\mathcal {O}$ while preserving non-sparsity for coefficients in $\mathcal {M}$.

When *d*=2, we have the design matrix,

3 $$ \mathbf{X}^{T}\mathbf{X}= n\left(\begin{array}{cc} 1 & \rho \\ \rho & 1 \\ \end{array} \right)  $$

with pairwise correlation *ρ*. We can show that the ridle estimates are

$$\begin{array}{@{}rcl@{}} \hat{\beta}_{1} &=& s_{1} \Big(|\hat{\beta}_{1}(\text{ols})| + \frac{\rho \lambda_{2}}{n-n\rho^{2}} \theta_{2} s_{1} \hat{\beta}_{2}(\text{ols}) - \theta_{1} \Big)^{+}, \\ \hat{\beta}_{2} \!&=& \!\left\{ \begin{array}{l} \theta_{2} \hat{\beta}_{2}(\text{ols}) + \frac{n\rho}{n+\lambda_{2}} s_{1} \theta_{1}, \\ \quad \quad \quad \quad \quad \,\,\,\, \text{if } \theta_{1} < |\hat{\beta}_{1}(\text{ols})| + \frac{\rho \lambda_{2}}{n-n\rho^{2}} \theta_{2} s_{1} \hat{\beta}_{2}(\text{ols}), \\ \frac{n+\lambda_{2}-n\rho^{2}}{(n+\lambda_{2})(1-\rho^{2})}\theta_{2} \hat{\beta}_{2}(\text{ols}) + \frac{n\rho}{n+\lambda_{2}}\hat{\beta}_{1}(\text{ols}), \quad \text{otherwise,} \end{array}\right. \end{array} $$

where $s_{1} = \text {sign} (\hat {\beta }_{1}(\text {ols}))$, *θ*_1_=*λ*_1_(*n*+*λ*_2_)/(2*n*(*n*+*λ*_2_−*n* *ρ* ^2^)), and *θ*_2_=*n*(1−*ρ* ^2^)/(*n*+*λ*_2_−*n* *ρ* ^2^). We see that the coefficient $\hat {\beta }_{1}$ can be thresholded to 0 with increasing *θ*_1_(*λ*_1_,*λ*_2_,*ρ*) and *θ*_2_(*λ*_2_,*ρ*) functions to increase (temper) the thresholding of $\hat {\beta }_{1}$ when $\rho s_{1} \hat {\beta }_{2}(\text {ols})$ is negative (positive). On the other hand, $\hat {\beta }_{2}$ converges to a weighted average of $\hat {\beta }_{1}(\text {ols})$ and $\hat {\beta }_{2}(\text {ols})$ without necessarily thesholding it to 0 as *θ*_1_ increases to $|\hat {\beta }_{1}(\text {ols})| + \rho \lambda _{2} \theta _{2} s_{1} \hat {\beta }_{2}(\text {ols})/(n-n\rho ^{2})$.

In the special case when *λ*_2_=0, the ridle is reduced to the $\mathcal {M}$-unpenalized lasso, with estimates $\mathcal {M}$-unpenalized lasso, with estimates $\hat {\beta }_{1}(\mathcal {M}-\text {unpenalized lasso}) = s_{1} (|\hat {\beta }_{1}(\text {ols})| - \theta _{1})^{+}$, $\hat {\beta }_{2} (\mathcal {M}-\text {unpenalized lasso}) = \hat {\beta }_{2}(\text {ols}) + s_{1} \rho \theta _{1}$ if $\theta _{1} < |\hat {\beta }_{1}(\text {ols})|$, and $\hat {\beta }_{2}(\mathcal {M}-\text {unpenalized lasso}) = \hat {\beta }_{2}(\text {ols}) + \rho \hat {\beta }_{1}(\text {ols})$ otherwise. Under multicollinearity when *ρ* is large, the OLS estimates are known to have large variability. In this case, the ridge is often employed to improve prediction accuracy by regulating variances. Compared with the ridle, the $\mathcal {M}$-unpenalized lasso that imposes no penalization on mandatory coefficients can be less effective in tempering the effects of multicollinearity. For example, when *ρ*=1 and *θ*_1_ is large, the $\mathcal {M}$-unpenalized lasso estimate for *β*_2_ is $\hat {\beta }_{2}(\text {ols}) + \hat {\beta }_{1}(\text {ols})$, such that the $\mathcal {M}$-unpenalized lasso can have larger prediction error than the OLS.

The lasso has the solution $\hat {\beta }_{j}(\text {lasso}) = s_{j} (|\hat {\beta }_{j}(\text {ols})| - \gamma)^{+}$ for *j*=1,2 and does not involve the correlation *ρ* when *d*=2 [4]. In contrast, ridge coefficients tend to be averaged with increasing correlation. This property helps ridge to reduce variances of its estimates and improve prediction accuracy when data is multicollinear [16]. The ridle estimates $(\hat {\beta }_{1},\hat {\beta }_{2})$ are also defined in terms of weighted averages of $\hat {\beta }_{1}(\text {ols})$ and $\hat {\beta }_{2}(\text {ols})$ according to correlation *ρ*. In the following, we will show via theoretical studies how this property can improve variable selection for ridle.

### Theoretical properties

In this section, we provide theoretical properties of the ridle estimator. These results are useful in providing a window to understanding the proposed method and a guide as to how the methods might perform in practice. Here, we use the sign-consistency approach [17] for theoretical derivations, which can provide results that are easy to interpret and relate to applications. More involved theoretical approaches, such as the asymptotic and non-asympotic oracle properties [7, 18], often rely on complex conditions that are difficult to interpret. Proofs for theoretical results in this section are provided in Additional file 1.

Without loss of generality, we assume that the true coefficients ${\beta }^{0} = (({\beta }^{0}_{(1)})^{T}, ({\beta }^{0}_{(2)})^{T}, ({\beta }^{0}_{(3)})^{T})^{T}$ are partitioned such that ${\beta }^{0}_{(1)}=\{\beta ^{0}_{j}: \beta ^{0}_{j} \neq 0~\text {and}~ j \in \mathcal {O} \}$, ${\beta }^{0}_{(2)}=\{\beta ^{0}_{j}: \beta ^{0}_{j} = 0~\text { and} ~j \in \mathcal {O} \}$, and ${\beta }^{0}_{(3)}=\{\beta ^{0}_{j}: j \in \mathcal {M} \}$. Let $\mathbf {C}^{n} = \mathbf {X}_{n}^{T} \mathbf {X}_{n} / n$ and $\tilde {\mathbf {C}}^{n} = \mathbf {C} + (\lambda _{2}/n) \mathbf {I}$. With the columns of **X**_*n*_ partitioned as *β* ^0^, **C** ^*n*^ has the expression

4 $$ \mathbf{C}^{n} = \left(\begin{array}{ccc} \mathbf{C}^{n}_{11} & \mathbf{C}^{n}_{12} & \mathbf{C}^{n}_{13} \\ \mathbf{C}^{n}_{21} & \mathbf{C}^{n}_{22} & \mathbf{C}^{n}_{23} \\ \mathbf{C}^{n}_{31} & \mathbf{C}^{n}_{32} & \mathbf{C}^{n}_{33} \\ \end{array} \right).  $$

We assume that

5 $$ \mathbf{C}^{n} \to \mathbf{C},  $$

where **C** is a positive definite matrix,

6 $$ \frac{1}{n} \max_{1\le i \le n} ((\mathbf{x}^{n}_{i})^{T} \mathbf{x}^{n}_{i}) \to 0,  $$

and

7 $$ \tilde{\mathbf{C}}^{n}_{33}~\text{and}~(\mathbf{C}^{n}_{11} - \mathbf{C}^{n}_{13} (\tilde{\mathbf{C}}^{n}_{33})^{-1} \mathbf{C}^{n}_{31})~\text{are invertible}.  $$

#### Asymptotic normality of the Ridle

##### **Theorem 1**

With (5) and (6), $\hat {\mathbf {\beta }}(\lambda _{1},\lambda _{2})$ satisfies the following for *λ*_1_,*λ*_2_>0 such that $\lambda _{1}/\sqrt {n} \to c_{1} < \infty $ and $\lambda _{2}/\sqrt {n} \to c_{2}$.

8 $$ \sqrt{n} (\hat{\mathbf{\beta}} - \mathbf{\beta}^{0}) \to_{d} \arg \min V(\mathbf{u})  $$

where

$$\begin{array}{@{}rcl@{}} V (\mathbf{u}) &=& \mathbf{u}^{T}\mathbf{C}\mathbf{u} - 2\mathbf{u}^{T}\mathbf{W}\\ &&+ c_{1} \sum_{j\in \mathcal{O}} (u_{j} \text{sign}(\beta_{j}^{0})I(\beta_{j}^{0} \neq 0) + |u_{j}|I(\beta_{j}^{0}=0))\\ &&+ 2 c_{2} \mathbf{u}^{T}_{(3)} \mathbf{\beta}^{0}_{(3)} \end{array} $$

and **W**∼*N*(**0**,*σ* ^2^ **C**).

Theorem 1 shows that the coefficients of mandatory covariates can contribute to the biasness of the ridle estimates. If the coefficients of variables in $\mathcal {M}$ are relatively large, then a small *c*_2_ is required to keep the bias low. On the other hand, when coefficients of variables in $\mathcal {M}$ are small, a wider spectrum of values of *c*_2_ can be chosen to improve prediction accuracy. Hence, we expect the ridle to perform the best when coefficients of mandatory variables tend to be small.

#### Variable selection consistency of the Ridle.

In this section, we provide sign consistency results for the ridle. We define the ridle estimates $\hat {\mathbf {\beta }}(\lambda _{1}, \lambda _{2})$ to be sign consistent if there exist *λ*_1_=*λ*_1_(*n*) and *λ*_2_=*λ*_2_(*n*) such that

9 $$ {\lim}_{n \to \infty} P(\text{sign}(\hat{\mathbf{\beta}}_{\mathcal{O}}(\lambda_{1}, \lambda_{2})) = \text{sign}({\mathbf{\beta}^{0}_{\mathcal{O}}})) = 1.  $$

Sign consistency, as a stronger condition, directly implies variable selection consistency.

With (5)-(7), we give the following conditions for sign consistency of the ridle estimator.

Sufficient condition: There exists *η*>0 such that

10 $$ |\mathbf{D}^{n}\text{sign}(\mathbf{\beta}^{0}_{(1)}) - \frac{2\lambda_{2}}{\lambda_{1}}(\mathbf{D}^{n}\mathbf{C}^{n}_{13} - \mathbf{C}^{n}_{23})(\tilde{\mathbf{C}}^{n}_{33})^{-1}\mathbf{\beta}^{0}_{(3)}| \le \mathbf{1}-\eta,  $$

where $\mathbf {D}^{n} =(\mathbf {C}^{n}_{21}-\mathbf {C}^{n}_{23}(\tilde {\mathbf {C}}^{n}_{33})^{-1}\mathbf {C}^{n}_{31}) (\mathbf {C}^{n}_{11}-\mathbf {C}^{n}_{13}(\tilde {\mathbf {C}}^{n}_{33})^{-1}\mathbf {C}^{n}_{31})^{-1}$.

Necessary condition:

11 $$ |\mathbf{D}^{n}\text{sign}(\mathbf{\beta}^{0}_{(1)}) - \frac{2\lambda_{2}}{\lambda_{1}}(\mathbf{D}^{n}\mathbf{C}^{n}_{13} - \mathbf{C}^{n}_{23})(\tilde{\mathbf{C}}^{n}_{33})^{-1}\mathbf{\beta}^{0}_{(3)}| < \mathbf{1}.  $$

##### **Theorem 2**

Under (5)-(7), $\hat {\mathbf {\beta }}(\lambda _{1},\lambda _{2})$ is sign consistent if condition (10) holds for *λ*_1_,*λ*_2_>0 such that *λ*_1_/*n*→0, $\lambda _{1}/\sqrt {n} \to \infty $, and *λ*_2_/*λ*_1_→*c*<*∞*.

##### **Theorem 3**

Under (5)-(7), $\hat {\mathbf {\beta }}(\lambda _{1},\lambda _{2})$ is sign consistent only if condition (11) holds for *λ*_1_,*λ*_2_>0 such that *λ*_2_/*n*→0.

##### **Remark 1**

Let $\mathbf {C}^{n}_{12}=\mathbf {C}^{n}_{23}=\mathbf {0}$. Then conditions (10) and (11) are satisfied with left-hand sides equal to **0**. Thus, the ridle estimator is sign consistent when predictors with nonzero coefficients are unrelated with predictors with zero coefficients.

##### **Remark 2**

Suppose $\mathbf {C}^{n}_{13}=\mathbf {C}^{n}_{23}=\mathbf {0}$. Then, conditions (10) and (11) become

$$\begin{aligned} &|\mathbf{C}^{n}_{21} (\mathbf{C}^{n}_{11})^{-1} \text{sign} (\mathbf{\beta}^{0}_{(1)})| < 1 -\eta \quad \text{and}\\ &|\mathbf{C}^{n}_{21} (\mathbf{C}^{n}_{11})^{-1} \text{sign} (\mathbf{\beta}^{0}_{(1)})| < 1, \end{aligned} $$

respectively. This is equivalent to the Irrepresentable conditions of [17] for lasso sign consistency. Thus, when variables in $\mathcal {M}$ are uncorrelated with those in $\mathcal {O}$, sign consistency for the ridle is equivalent to that of the lasso for predictors in $\mathcal {O}$.

##### **Remark 3**

Consider performing the lasso on predictors in both $\mathcal {O}$ and $\mathcal {M}$. The following Irrepresentable conditions are derived in Zhao and Yu (2006) for the lasso [17],

12 $$\begin{array}{*{20}l}   \text{(sufficient)} \qquad |\mathbf{D}^{n}_{1} \text{sign}(\mathbf{\beta}^{0}_{(1)}) + \mathbf{D}^{n}_{2} \text{sign}(\mathbf{\beta}^{0}_{(3)})| &< 1 - \eta \end{array} $$

13 $$\begin{array}{*{20}l}   \text{(necessary)} \qquad |\mathbf{D}^{n}_{1} \text{sign}(\mathbf{\beta}^{0}_{(1)}) + \mathbf{D}^{n}_{2} \text{sign}(\mathbf{\beta}^{0}_{(3)})| &< 1 \end{array} $$

where $\mathbf {D}^{n}_{1} = \mathbf {C}^{n}_{21} - \mathbf {C}^{n}_{23} (\mathbf {C}^{n}_{33})^{-1} \mathbf {C}^{n}_{31}) (\mathbf {C}^{n}_{11} - \mathbf {C}^{n}_{13} (\mathbf {C}^{n}_{33})^{-1} \mathbf {C}^{n}_{31})^{-1}$ and $\mathbf {D}^{n}_{2} = (\mathbf {C}^{n}_{23} - \mathbf {C}^{n}_{21} (\mathbf {C}^{n}_{11})^{-1} \mathbf {C}^{n}_{13}) (\mathbf {C}^{n}_{33} - \mathbf {C}^{n}_{31} (\mathbf {C}^{n}_{11})^{-1} \mathbf {C}^{n}_{13})^{-1}$. Compared with the lasso, the ridle conditions in (10) and (11) involve the parameter *λ*_2_ that can allow it to more easily satisfy sign consistency conditions with suitable choices of *λ*_2_. Consider a toy example to better understand the general results through a simple scenario. If $\mathbf {C}^{n}_{23}\neq \mathbf {0}$ and $\mathbf {C}^{n}_{12}=\mathbf {C}^{n}_{13}=\mathbf {0}$, then the left-hand sides of conditions (10) and (11) for the ridle become

$$|2(\lambda_{2}/\lambda_{1}) \mathbf{C}^{n}_{23} (\tilde{\mathbf{C}^{n}_{33}})^{-1} \mathbf{\beta}^{0}_{(3)}|, $$

whereas the left-hand sides of conditions (12) and (13) for the lasso are

$$|\mathbf{C}^{n}_{23} (\mathbf{C}^{n}_{33})^{-1} \text{sign} (\mathbf{\beta}^{0}_{(3)})|. $$

Here, the ridle is sign consistent for *λ*_2_/*λ*_1_ sufficiently small, but the lasso is not if the elements of $\mathbf {C}^{n}_{23}$ are large or $\mathbf {C}^{n}_{33}$ is nearly singular. Thus, we expect that the ridle may perform better than lasso using predictors from both $\mathcal {O}$ and $\mathcal {M}$ in terms of model selection if predictors in $\mathcal {M}$are correlated with the irrelevant predictors in $\mathcal {O}$ or the predictors in $\mathcal {M}$ are highly correlated among themselves.

##### **Remark 4**

When *λ*_2_=0, the ridle is reduced to the $\mathcal {M}$-unpenalized lasso. In this case, as no penalization is involved on mandatory coefficients, sign consistency conditions can be trivially obtained as

14 $$  \left|\mathbf{D}^{n}_{1} \text{sign}\left(\mathbf{\beta}^{0}_{(1)}\right)\right| < 1 - \eta \qquad \text{and} \qquad \left|\mathbf{D}^{n}_{1} \text{sign}\left(\mathbf{\beta}^{0}_{(1)}\right)\right| < 1,  $$

where $D_{1}^{n}$ is as defined following conditions (12) and (13) for the lasso. Compared with the $\mathcal {M}$-unpenalized lasso, the ridle composes of an additional offsetting factor $({2\lambda _{2}}/{\lambda _{1}})(\mathbf {D}^{n}\mathbf {C}^{n}_{13} - \mathbf {C}^{n}_{23})(\tilde {\mathbf {C}}^{n}_{33})^{-1}\mathbf {\beta }^{0}_{(3)}$ in conditions (10) and (11) that allows sign consistency inequalities to be more easily satisfied. For example, if $\mathbf {C}^{n}_{23} = \mathbf {0}$, $\mathbf {C}^{n}_{12}\neq \mathbf {0}$, and $\mathbf {C}^{n}_{13}\neq \mathbf {0}$, then the left-hand sides of conditions (10) and (11) for the ridle become

$$\left|\mathbf{D}^{n} \text{sign}\left(\mathbf{\beta}^{0}_{(1)}\right) - 2\left(\lambda_{2}/\lambda_{1}\right) \mathbf{D}^{n} \mathbf{C}^{n}_{13} \left(\tilde{\mathbf{C}^{n}_{33}}\right)^{-1} \mathbf{\beta}^{0}_{(3)}\right|. $$

In this case, if $|\mathbf {D}^{n}_{1} \text {sign}(\mathbf {\beta }^{0}_{(1)}) | \ge 1$, sign consistency conditions for the $\mathcal {M}$-unpenalized lasso in (14) are violated, whereas sign consistency conditions for the ridle may still be satisfied with suitably chosen *λ*_2_.

##### **Remark 5**

When the mandatory covariates are irrelevant, $\mathbf {\beta }^{0}_{(3)}=\mathbf {0}$, the offsetting terms in (10) and (11) for ridle sign consistency would vanish. Indeed, with *λ*_2_/*n*→0, ridle sign consistency conditions are equivalent to those of both the lasso and $\mathcal {M}$-unpenalized lasso. However, this does not mean that the methods will perform similarly under finite samples. We will examine their finite-sample performances in the *Analysis of Simulated Data* in the “Results” section.

### Efficient algorithm

We provide an efficient algorithm for computing the ridle. Programming code, written in Fortran, and its R-language wrapper for the algorithm described in this section are freely available online at http://sites.google.com/site/zhongyindaye/software.

The ridle (1) minimizes over an objective function with convex and separable penalties. This allows us to employ the coordinate descent strategy [19–21] to compute for the ridle. In the coordinate descent, we update first for all coefficients of mandatory variables $\mathbf {\beta }_{\mathcal {M}}$ and, then, each optional coefficient $\beta _{j} \in \mathcal {O}$, one at a time. This is iterated till practical convergence is reached. The algorithm is further sped up by iterating only through the mandatory and active set till convergence before updating all variables. We provide the coordinate descent updating equations as the following,

15 $$\begin{array}{@{}rcl@{}}   \mathbf{\beta}_{\mathcal{M}} &\leftarrow& \left(\mathbf{X}_{\mathcal{M}}^{T} \mathbf{X}_{\mathcal{M}} + \lambda_{2} \mathbf{I}\right)^{-1} \mathbf{X}^{T}_{\mathcal{M}} \left(\mathbf{y} - \mathbf{X}_{\mathcal{O}} \mathbf{\beta}_{\mathcal{O}}\right) \end{array} $$

16 $$\begin{array}{@{}rcl@{}}  \beta_{j} \!&\leftarrow&\! \frac{s_{j}}{\|\mathbf{x}_{j}\|^{2}}\! \left(\left| \mathbf{x}^{T}_{j} \!\left(\!\mathbf{y} \!- \!\sum_{k \neq j} \mathbf{x}_{k} \beta_{k}\right)\!\right|\! -\! \frac{\lambda_{1}}{2} \!\right)_{+} \!\!\text{for}~j\in\mathcal{O}, \end{array} $$

where $s_{j} = \text {sign}(\mathbf {x}^{T}_{j} (\mathbf {y} - \sum _{k \neq j} \mathbf {x}_{k} \beta _{k}))$. Maximum value for *λ*_1_ is $\lambda _{1}^{max} = 2 \max _{j \in \mathcal {O}} | \mathbf {y} - \mathbf {X}_{\mathcal {M}} \hat {\mathbf {\beta }}_{\mathcal {M},0} |$, where $\hat {\mathbf {\beta }}_{\mathcal {M},0} = (\mathbf {X}_{\mathcal {M}}^{T} \mathbf {X}_{\mathcal {M}} + \lambda _{2} \mathbf {I})^{-1} \mathbf {X}^{T}_{\mathcal {M}} \mathbf {y}$ are initial estimates for coefficients of mandatory covariates. The matrix inverse in (15) can be computed efficiently by taking the inverse of individual eigenvalues added to *λ*_2_ after an initial singular value decomposition of $\mathbf {X}_{\mathcal {M}}^{T} \mathbf {X}_{\mathcal {M}}$.

## Results

### Analysis of simulated data

We evaluate the performances of the ridle via simulation studies. We examine effects of having different magnitudes of coefficients, correlations between mandatory and irrelevant predictors, and degrees of multicollinearity among mandatory covariates. We compare the ridle to the ridge, lasso, elastic net, and the lasso and elastic net without penalization on the mandatory covariates. We use the R package *glmnet 2.0* to compute for the lasso and elastic net, where the penalization on mandatory covariates is specified using the penalty.factor option.

In each example, we simulate 200 times from the true model, **y**=**X** *β*+*σ* ***ε***, where ***ε***∼**N**(**0**,**I**). We use *n*=50 number of observations and *p*=250 predictors. Tuning parameters are estimated using 5-fold cross-validation. We measure prediction accuracy using the relative prediction error, $rpe=(\hat {\beta }-{\beta })^{T} \boldsymbol {\Sigma } (\hat {\beta }-{\beta })/ \sigma ^{2}$, where ***Σ*** is the population covariance matrix. Further, we examine variable selection performances using sensitivity, specificity, and *g*-measure. Sensitivity and specificity are, respectively, the marginal proportions of selecting relevant variables and discarding irrelevant variables correctly. In other words, sensitivity is the proportion of true positives among all relevant variables, whereas specificity is the proportion of true negatives among all irrelevant variables. The false positive rate is equal to 1 - specificity. As proportions, sensitivity and specificity allow intuitive comparisons across simulation settings with varying numbers of relevant and irrelevant variables in high-dimensional variable selection. We examine overall variable selection performances using the *g*-measure, $\sqrt {sensitivity*specificity}$. A *g*-measure close to 1 indicates accurate variable selection, whereas a *g*-measure close to 0 implies that few relevant variables or too many irrelevant variables are selected, or both. In Tables 1, 2, 3 and 4, we report medians and bootstrapped standard deviations of medians out of 500 re-samplings, in parentheses. Further, we boldface, as top measurements, the smallest rpe and two largest g-measures in each case.

**Table 1:** Simulation example 1: effect of signal strengths

	Method	*rpe*	*g*-measure	Sensitivity	Specificity
*β*_0_=0.5	Ridge	1.008 (0.009)			
	Lasso	1.004 (0.018)	0.582 (0.009)	0.350 (0.018)	0.957 (0.006)
	Elastic net	0.923 (0.020)	0.676 (0.007)	0.600 (0.041)	0.848 (0.023)
	$\mathcal {M}$-unpenalized lasso	0.675 (0.028)	**1.000** (0.000)	1.000 (0.000)	1.000 (0.000)
	$\mathcal {M}$-unpenalized elastic net	0.697 (0.026)	**1.000** (0.001)	1.000 (0.000)	1.000 (0.002)
	Ridle	**0.281** (0.016)	**0.998** (0.001)	1.000 (0.000)	0.996 (0.002)
*β*_0_=1.5	Ridge	6.549 (0.056)			
	Lasso	3.300 (0.083)	0.839 (0.005)	0.750 (0.017)	0.926 (0.003)
	elastic net	3.230 (0.118)	0.853 (0.004)	0.900 (0.008)	0.850 (0.005)
	$\mathcal {M}$-unpenalized lasso	0.691 (0.023)	**1.000** (0.000)	1.000 (0.000)	1.000 (0.000)
	$\mathcal {M}$-unpenalized elastic net	0.701(0.028)	**1.000** (0.001)	1.000 (0.000)	1.000 (0.001)
	Ridle	**0.473** (0.014)	**0.998** (0.001)	1.000 (0.000)	0.996 (0.002)
*β*_0_=3	Ridge	24.559 (0.317)			
	Lasso	8.074 (0.433)	0.908 (0.005)	0.900 (0.013)	0.935 (0.002)
	Elastic net	6.735 (0.339)	0.903 (0.002)	0.950 (0.013)	0.852 (0.003)
	$\mathcal {M}$-unpenalized lasso	0.676 (0.032)	**1.000** (0.000)	1.000 (0.000)	1.000 (0.000)
	$\mathcal {M}$-unpenalized elastic net	0.725 (0.030)	**1.000** (0.000)	1.000 (0.000)	1.000 (0.001)
	Ridle	**0.605** (0.025)	**0.998** (0.001)	1.000 (0.000)	0.996 (0.002)

The $\mathcal {M}$-unpenalized lasso and $\mathcal {M}$-unpenalized elastic net were performed without penalization on the mandatory covariates

*n*=50, *p*=250, $|\mathcal {M}|=20$. The smallest *rpe* and largest two *g*-measures are boldfaced

**Table 2:** Simulation example 2: effect of correlation between mandatory and irrelevant predictors

	Method	*rpe*	*g*-measure	Sensitivity ($\mathcal {M}$)	Sensitivity ($\mathcal {O}$)	Specificity ($\mathcal {O}$)
*ρ*_0_=0.25	Ridge	1.671 (0.012)				
	Lasso	1.911 (0.022)	0.383 (0.034)	0.100 (0.032)	0.200 (0.028)	0.975 (0.008)
	Elastic net	1.744 (0.019)	0.585 (0.015)	0.400 (0.054)	0.600 (0.050)	0.835 (0.036)
	$\mathcal {M}$-unpenalized lasso	1.741 (0.028)	0.742 (0.012)	1.000 (0.000)	0.200 (0.037)	0.938 (0.003)
	$\mathcal {M}$-unpenalized elastic net	1.657 (0.017)	**0.757** (0.008)	1.000 (0.000)	0.500 (0.064)	0.833 (0.022)
	Ridle	**1.492** (0.031)	**0.773** (0.006)	1.000 (0.000)	0.200 (0.048)	0.931 (0.006)
*ρ*_0_=0.5	Ridge	1.807 (0.014)				
	Lasso	2.045 (0.035)	0.571 (0.013)	0.300 (0.046)	0.400 (0.039)	0.925 (0.007)
	Elastic net	1.773 (0.034)	0.667 (0.008)	0.600 (0.014)	0.800 (0.048)	0.756 (0.020)
	$\mathcal {M}$-unpenalized lasso	1.922 (0.044)	0.794 (0.003)	1.000 (0.000)	0.400 (0.047)	0.929 (0.004)
	$\mathcal {M}$-unpenalized elastic net	1.729 (0.040)	**0.796** (0.007)	1.000 (0.000)	0.700 (0.048)	0.785 (0.022)
	Ridle	**1.438** (0.057)	**0.852** (0.006)	1.000 (0.000)	0.600 (0.049)	0.900 (0.004)
*ρ*_0_=0.75	Ridge	1.564 (0.022)				
	Lasso	1.365 (0.029)	0.684 (0.008)	0.400 (0.032)	0.600 (0.012)	0.900 (0.003)
	Elastic net	1.237 (0.030)	0.745 (0.005)	0.700 (0.048)	0.900 (0.011)	0.775 (0.014)
	$\mathcal {M}$-unpenalized lasso	1.423 (0.037)	0.839 (0.005)	1.000 (0.000)	0.700 (0.026)	0.904 (0.006)
	$\mathcal {M}$-unpenalized elastic net	1.310 (0.041)	**0.847** (0.005)	1.000 (0.000)	0.800 (0.012)	0.840 (0.008)
	Ridle	**0.886** (0.029)	**0.875** (0.003)	1.000 (0.000)	0.700 (0.038)	0.908 (0.003)

The $\mathcal {M}$-unpenalized lasso and $\mathcal {M}$-unpenalized elastic net were performed without penalization on the mandatory covariates. *g*-measure is estimated from all predictors. Sensitivity ($\mathcal {M}$) is computed in terms of the mandatory variables only, whereas sensitivity ($\mathcal {O}$) and specificity ($\mathcal {O}$) are computed in terms of the optional variables only

*n*=50, *p*=250, $|\mathcal {M}|=10$. The smallest *rpe* and largest two *g*-measures are boldfaced

**Table 3:** Simulation example 3: effect of multicollinearity among mandatory covariates

	Method	*rpe*	*g*-measure	Sensitivity ($\mathcal {M}$)	Sensitivity ($\mathcal {O}$)	Specificity ($\mathcal {O}$)
*ρ*=0.75	Ridge	6.353 (0.022)				
	Lasso	4.649 (0.167)	0.802 (0.011)	0.800 (0.000)	0.700 (0.048)	0.908 (0.004)
	Elastic net	4.410 (0.128)	0.804 (0.005)	1.000 (0.009)	0.700 (0.006)	0.858 (0.006)
	$\mathcal {M}$-unpenalized lasso	4.776 (0.260)	**0.829** (0.005)	1.000 (0.000)	0.700 (0.031)	0.902 (0.007)
	$\mathcal {M}$-unpenalized elastic net	5.402 (0.190)	0.823 (0.006)	1.000 (0.000)	0.700 (0.013)	0.871 (0.009)
	Ridle	**2.699** (0.152)	**0.893** (0.007)	1.000 (0.000)	0.900 (0.048)	0.904 (0.004)
*ρ*=0.9	Ridge	6.270 (0.026)				
	Lasso	4.914 (0.148)	0.784 (0.010)	0.600 (0.089)	0.700 (0.036)	0.908 (0.004)
	Elastic net	4.336 (0.135)	0.816 (0.005)	0.800 (0.092)	0.700 (0.018)	0.867 (0.008)
	$\mathcal {M}$-unpenalized lasso	6.992 (0.337)	**0.828** (0.008)	1.000 (0.000)	0.700 (0.031)	0.902 (0.006)
	$\mathcal {M}$-unpenalized elastic net	7.245 (0.237)	0.827 (0.005)	1.000 (0.000)	0.700 (0.045)	0.860 (0.011)
	Ridle	**3.000** (0.214)	**0.890** (0.006)	1.000 (0.000)	0.800 (0.045)	0.900 (0.004)
*ρ*=0.99	Ridge	6.231 (0.031)				
	Lasso	7.322 (0.200)	0.745 (0.005)	0.400 (0.000)	0.700 (0.000)	0.913 (0.003)
	Elastic net	5.003 (0.155)	0.804 (0.006)	0.800 (0.049)	0.700 (0.019)	0.883 (0.006)
	$\mathcal {M}$-unpenalized lasso	36.214 (2.064)	0.824 (0.006)	1.000 (0.000)	0.700 (0.046)	0.904 (0.005)
	$\mathcal {M}$-unpenalized elastic net	33.583 (2.197)	**0.830** (0.004)	1.000 (0.010)	0.700 (0.045)	0.867 (0.010)
	Ridle	**4.193** (0.343)	**0.890** (0.005)	1.000 (0.000)	0.800 (0.029)	0.904 (0.004)

The $\mathcal {M}$-unpenalized lasso and $\mathcal {M}$-unpenalized elastic net were performed without penalization on the mandatory covariates. *g*-measure is estimated from all predictors. Sensitivity ($\mathcal {M}$) is computed in terms of the mandatory variables only, whereas sensitivity ($\mathcal {O}$) and specificity ($\mathcal {O}$) are computed in terms of the optional variables only

*n*=50, *p*=250, $|\mathcal {M}|=5$. The smallest *rpe* and largest two *g*-measures are boldfaced

**Table 4:** Simulation example 4: mandatory covariates are irrelevant

	Method	*rpe*	*g*-measure	Specificity ($\mathcal {M}$)	Sensitivity ($\mathcal {O}$)	Specificity ($\mathcal {O}$)
*ρ*_0_=0.25	Ridge	**1.671** (0.012)				
	Lasso	1.911 (0.022)	**0.383** (0.034)	1.000 (0.000)	0.200 (0.028)	0.975 (0.008)
	Elastic net	1.744 (0.019)	**0.585** (0.015)	0.600 (0.053)	0.600 (0.050)	0.835 (0.036)
	$\mathcal {M}$-unpenalized lasso	2.357 (0.032)	0.215 (0.103)	0.000 (0.000)	0.050 (0.024)	0.995 (0.003)
	$\mathcal {M}$-unpenalized elastic net	2.210 (0.034)	0.308 (0.054)	0.000 (0.000)	0.525 (0.065)	0.732 (0.057)
	Ridle	1.854 (0.012)	0.309 (0.029)	0.000 (0.000)	0.100 (0.024)	0.982 (0.005)
*ρ*_0_=0.5	Ridge	1.807 (0.014)				
	Lasso	2.045 (0.035)	**0.571** (0.013)	0.800 (0.006)	0.400 (0.039)	0.925 (0.007)
	Elastic net	**1.773** (0.034)	**0.667** (0.008)	0.500 (0.048)	0.800 (0.048)	0.756 (0.020)
	$\mathcal {M}$-unpenalized lasso	2.242 (0.023)	0.299 (0.035)	0.000 (0.000)	0.100 (0.021)	0.982 (0.004)
	$\mathcal {M}$-unpenalized elastic net	2.080 (0.028)	0.305 (0.094)	0.000 (0.000)	0.550 (0.072)	0.700 (0.079)
	Ridle	1.801 (0.039)	0.528 (0.032)	0.000 (0.000)	0.300 (0.038)	0.943 (0.005)
*ρ*_0_=0.75	Ridge	1.564 (0.022)				
	Lasso	1.365 (0.029)	**0.684** (0.008)	0.700 (0.041)	0.600 (0.012)	0.900 (0.003)
	Elastic net	**1.237** (0.030)	**0.745** (0.005)	0.300 (0.046)	0.900 (0.011)	0.775 (0.014)
	$\mathcal {M}$-unpenalized lasso	1.747 (0.043)	0.428 (0.003)	0.000 (0.000)	0.200 (0.000)	0.964 (0.003)
	$\mathcal {M}$-unpenalized elastic net	1.662 (0.043)	0.514 (0.016)	0.000 (0.000)	0.350 (0.023)	0.900 (0.015)
	Ridle	1.253 (0.042)	0.596 (0.017)	0.000 (0.000)	0.400 (0.026)	0.945 (0.003)

The $\mathcal {M}$-unpenalized lasso and $\mathcal {M}$-unpenalized elastic net were performed without penalization on the mandatory covariates. *g*-measure is estimated from all predictors. specificity ($\mathcal {M}$) is computed in terms of the mandatory variables only, whereas sensitivity ($\mathcal {O}$) and specificity ($\mathcal {O}$) are computed in terms of the optional variables only

*n*=50, *p*=250, $|\mathcal {M}|=10$. The smallest *rpe* and largest two *g*-measures are boldfaced

#### Example 1 (Effect of signal strengths)

This example has *β*_*j*_=*β*_0_ for *j*∈{1,…,10,21,…,30} and *β*_*j*_=0 otherwise. Predictors are generated from **X**∼*N*(**0**,**Σ**) where *Σ*_*ij*_=0.5^|*i*−*j*|^. *σ*=3. We assume the mandatory covariates to be comprised of the relevant variables so that $\mathcal {M}= \{1,\ldots,10,21,\ldots,30\}$.

Table 1 displays prediction accuracy and variable selection performances for this example. First of all, by utilizing a priori information on mandatory covariates, the ridle has significantly smaller *rpe*’s than those of the ridge, lasso and elastic net with or without penalization on mandantory covariates. Additionally, the ridle has larger *g*-measure than those of the lasso and elastic net and similar *g*-measure with the mandantory-unpenalized lasso and elastic-net method. Sensitivity for the lasso and elastic net decreases dramatically as the signal strength weakens or *β*_0_ becomes smaller. On the other hand, specificity for the lasso decreases while increasing for elastic net when *β*_0_ becomes larger. Furthermore, the lasso and elastic net without penalization on mandantory variables outperforms the lasso and elastic net with penaliztion on the mandantory variables in terms of both prediction accuracy and variable selection. These suggest that, even though the elastic net does a better job than the lasso in terms of prediction accuracy, both methods may not be able to distinguish well between mandatory and irrelevant variables, and incorporating a priori knowledge on mandatory covariates can yield significant improvements.

#### Example 2 (Effect of correlation between mandatory and irrelevant predictors)

In this example, we have *β*_*j*_=2 for *j*∈{2*k*:*k*=1,…,10}, *β*_*j*_=1.5 for *j*∈{2*k*:*k*=11,…,20}, and *β*_*j*_=0 otherwise. Predictors are generated from **X**∼*N*(**0**,**Σ**) where each element $\Sigma _{ij}= \rho _{0}^{|i-j|}$. Thus, relevant predictors are interspersed with irrelevant ones, to which they are correlated. Further, we assume $\mathcal {M}= \{ 2k: k=11, \ldots, 20 \}$ and *σ*=6. $\Sigma _{ij} = \rho _{0}^{|i-j|}$ presents an autocorrelated dependence structure, such that a variable **x**_*j*_ has a correlation of *ρ*_0_ with its immediate neighbors **x**_*j*−1_ and **x**_*j*+1_ for 1<*j*<*p*. When *ρ*_0_ is large, each variable is highly correlated with its immediate neighbors, resulting in multicollinearity.

Table 2 presents prediction accuracy and variable selection performances for this example. The ridle performs the best in terms of *rpe*’s. When *ρ*_0_ is large at 0.75, mandatory covariates are strongly correlated with some of the optional variables, and the $\mathcal {M}$-unpenalized lasso performs the worst in terms of prediction accuracy, except that of the ridge. This corroborates comments in *Two-Predictor Case* of “Methods” section that suggest the $\mathcal {M}$-unpenalized lasso can have large prediction errors under multicollinearity. Further, the ridle performs the best in terms of *g*-measures for overall variable selection in all scenarios.

#### Example 3 (Effect of multicollinearity among mandatory covariates)

Here, we have *β*_*j*_=3 for *j*∈{1,…,5}, *β*_*j*_=1.5 for *j*∈{6,…,10}, *β*_*j*_=2 for *j*∈{16,…,20}, and *β*_*j*_=0 otherwise. We set *σ*=3 and assume $\mathcal {M}= \{16, \ldots, 20\}$. Let *Z*∼*N*(0,1) and **ε**_*x*_∼*N*(0,1). We generate predictors as $\mathbf {x}_{j} = Z + \sqrt {(1-\rho)/\rho } \, \mathbf {\epsilon }$ for $j \in \mathcal {M}$ and **x**_*j*_∼*N*(0,1) otherwise. This creates correlations of *ρ* among the mandatory covariates.

In Table 3, we see that sensitivity ($\mathcal {M}$) decreases for the lasso and elastic net as *ρ* increases. Additionally, the lasso and elastic net without penalization on mandantory variables have identical sensitivity ($\mathcal {M}$) with the ridle. Furthermore, prediction error for the lasso without penalization on mandatory covariates increases dramatically as *ρ* increases, whereas the ridle has the lowest *rpe*’s. This corroborates Remark 3 of *Variable Selection Consistency of the Ridle* in “Methods” section, which suggests that the ridle may outperform the lasso when mandatory variables are highly correlated among themselves.

#### Example 4 (Mandatory covariates are irrelevant)

We repeat the simulation setting from example 2, but with the mandatory covariates defined as $\mathcal {M}= \{ 2k-1: k=1, \ldots, 10 \}$. In this case, the mandatory covariates are irrelevant.

Table 4 presents prediction accuracy and variable selection performances for this scenario. The ridle underperforms the elastic net but outperforms all other variable selection methods in terms of prediction accuracy. Indeed, the ridle has significantly smaller *rpe*’s compared with the $\mathcal {M}$-unpenalized lasso and $\mathcal {M}$-unpenalized elastic net. Moreover, the ridle underperforms both the lasso and elastic net in terms of *g*-measures for overall variable selection. However, ridle outperforms the $\mathcal {M}$-unpenalized lasso and $\mathcal {M}$-unpenalized elastic net at *ρ*_0_=0.5 and *ρ*_0_=0.75, when the irrelevant mandatory covariates are moderately and highly correlated, respectively, with some of the relevant optional variables. These suggest that the ridle, although not designed to exclude mandatory covariates when they are irrelevant, can be more advantageous than related methods that include mandatory covariates, as the ridle penalizes coefficients of irrelevant mandatory covariates towards, although not equal to, 0 with the ridge penalty.

### Gene expression analysis on histologic grades of breast cancer

Histologic grades are an important determinant of the aggressive potential of breast cancers and are of practical importance in the assessment and choice of treatment options. In this section, we apply our proposed method on a microarray gene expression dataset to determine genes that may be predictive of breast tumor histologic grade [22]. In this experiment, 251 frozen tumor tissure were collected from primary breast cancer patients and more than 12,000 genes were assayed on 251 subjects. We removed 2 subjects with missing outcomes and performed our analysis with the remaining 249 observations. Clinicopathological variables, such as ER status, PgR status, age and tumor size, measured at diagnosis, were obtained from patient records. Histologic grades are based on the widely used Nottingham Histologic Score system for prognosis of breast cancer [23]. There are three factors that pathologists consider in this scoring system: cell differentiation, nuclear features and mitotic activity [24]. Considerations of these factors allow the Nottingham Prognostic Index (NPI) to provide comprehensive prognosis of breast cancers. The three factors are each assigned a score from 1–3 based on clinical observations. A tumor is assigned a score of 1, 2, or 3 for cell differentiation if >75%, 10%-75%, or <10% of tumor area form glandular structures, respectively. A tumor has a score of 1, 2, or 3 for nuclear features if nuclei have little increase in size, larger than normal breast epithelial cells, or prominent nucleoli with occasionally very large sizes, respectively. Further, breast tumors have scores of 1, 2, or 3 for mitotic activity if ≤7, 8–14, or ≥15 mitoses per 10 high power microscopic fields are observed, respectively. Overall tumor grades are obtained by summing the scores for the three factors. Breast tumors with total scores of 3–5, 6–7, and 8–9 are assigned with tumor grades 1 (low), 2 (intermediate), and 3 (high), respectively, that represent the aggressive potential of breast tumors. The higher the grade is, the more likely it will spread or become aggressive. This dataset is available at the NCBI Gene Expression Omnibus (GEO) repository with GEO accession: gse3294. We focused our analysis on 430 genes from several well-known cancer-related pathways: PI3K [25, 26], p53 signaling [27–29], VEGF [30, 31], Hedgehog signaling [32, 33], ErbB signaling [34, 35], Ras signaling [36, 37] and Ion-channel family [38, 39].

Significant genes are selected as predictors of breast tumor grade along the 7 pathways by utilizing a sparse regression approach [40, 41]. In this strategy, tumor grade is regressed upon both the 4 clinicopathological covariates (ER status, PgR status, age and tumor size) and 430 gene expression levels, and significant predictor to tumor grade based on clinical covariates and genes are identified if they are retained in sparse regression analysis. We applied the ridle to perform variable selection on gene expression levels while conditioning on the 4 clinicopathological variables that we incorporated as mandatory covariates. We further compared our results with those from the ridge, lasso, elastic net, and lasso and elastic net without penalization on the 4 mandatory covariates.

Table 5 presents the numbers of genes and clinical covariates selected and the mean-squared error (*MSE*) using the strategies of ridge, lasso, and elastic net on both the 4 clinicopathological variables and 430 gene expression levels. Moreover, we present results from the lasso and elastic net without penalization on the 4 mandatory covariates, in addition to the ridle. The ridge has the largest *MSE*, suggesting the need for sparse regression. The lasso and elastic net performed similarly with the lasso and elastic net without penalization on mandatory covariates, respectively, in terms of the *MSE*. On the other hand, the ridle performed the best with the smallest *MSE* among all methods. This suggests that the ridle may be advantageous in predicting histologic grades of breast cancer.

**Table 5:** Gene expression analysis on histologic grades of breast cancer

	No. selected $\mathcal {M}$	No. selected $\mathcal {O}$	*MSE*
Ridge	4	430	0.487
Lasso	2	19	0.260
Elastic net	2	14	0.286
$\mathcal {M}$-unpenalized lasso	4	21	0.257
$\mathcal {M}$-unpenalized elastic net	4	7	0.296
Ridle	4	24	**0.239**

The $\mathcal {M}$-unpenalized lasso and $\mathcal {M}$-unpenalized elastic net were performed without penalization on the mandatory covariates. The elastic net and $\mathcal {M}$-unpenalized elastic net are built with alpha=0.2575 and alpha=0.8462, respectively, selected by cross-validation. Numbers of selected mandatory covariates $\mathcal {M}$ and optional variables $\mathcal {O}$, and mean-squared error (*MSE*) are shown. Smallest *MSE* is boldfaced

**Fig. 2:**
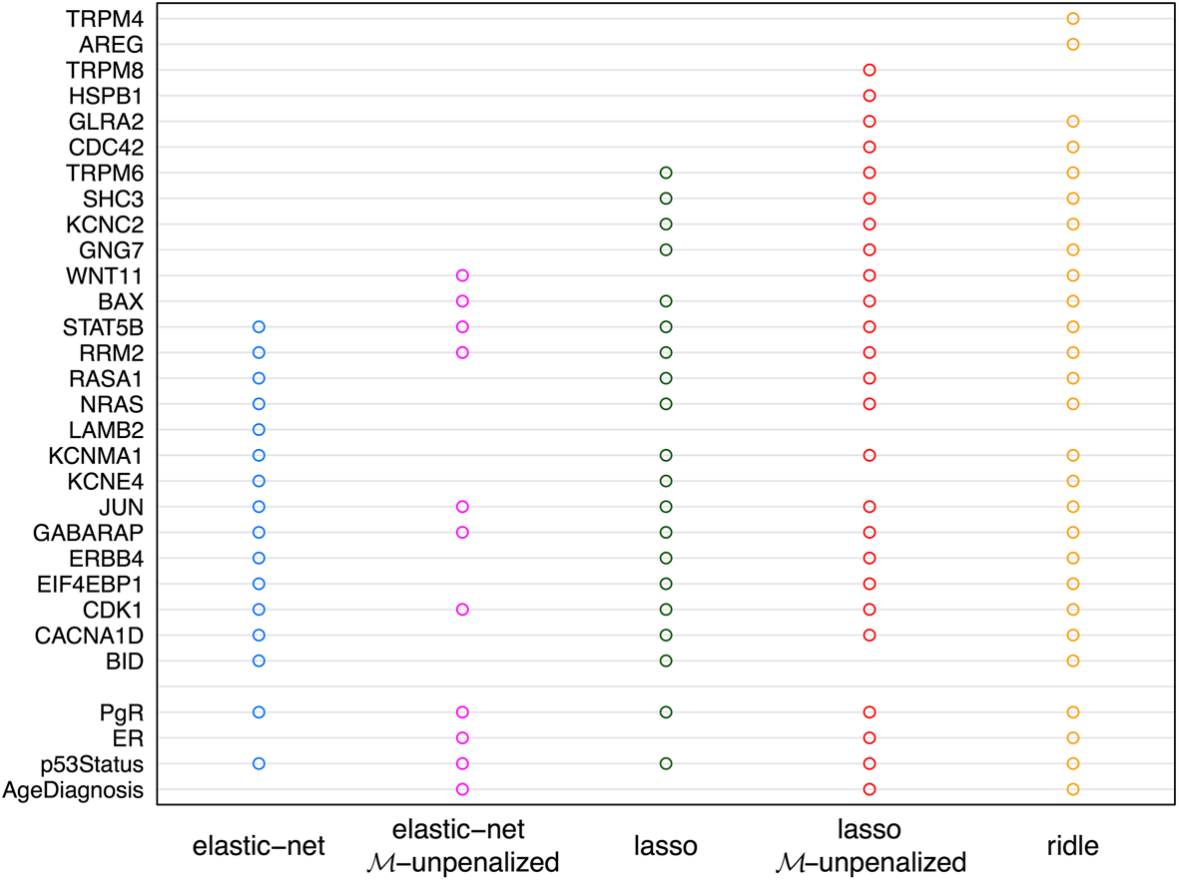
Selection of genes and clinicopathological variables. PgR, ER, p53Status, and AgeDiagnosis are clinicopathological covariates, whereas all others are genes. The $\mathcal {M}$-unpenalized lasso and $\mathcal {M}$-unpenalized elastic net were performed without penalization on the clinicopathological variables as mandatory covariates

Figure 2 depicts the genes selected via sparse regression methods. The ridle encompasses all of the genes selected by the lasso and elastic net with penalization on mandatory covariates, and nearly all of the genes selected by the elastic net (except 1 gene) and lasso without penalization on mandatory covariates (except 2 genes). Two genes are selected only by the ridle: the AREG and TRPM4 genes, which belong to the ErbB signaling pathway and ion-channel family, respectively.

Cells are continuously exposed to stimuli from paracrine and endocrine factors. It is essential that the extracellular signals are interpreted by cell correctly in order to facilitate proper proliferative response. The ErbB family belongs to receptors of the tyrosine kinase family and plays pivotal roles in this process [34]. Members of the ErbB signaling pathway have been suggested as potential therapeutic targets [42]. Initial studies have also suggested that expression levels of AREG (amphiregulin) are associated with larger and more aggressive tumors through cell proliferation [43, 44]. Only the ridle identified AREG as predictive of histologic grades of breast cancers.

The other gene selected only by the ridle is TRPM4 from the ion-channel family. Researches over the past few years have shown that ion channels are involved in the progression and pathology of a myriad of human cancers [39, 45, 46]. In addition, ion channels are known to play critical roles in gene expression, hormone secretion, cell volume regulation, and cell proliferation [47, 48]. The expression levels of ion-channel genes, including TRPM4, have been found to be predictive of and significantly associated with tumor progression [38].

Breast cancer is known to be highly correlated with hormone secretion. Breast tumors that are ER or PgR-positive are much more likely to respond to hormone therapy than tumors that are negative. Many of these may not be related to histologic grades of breast cancer. For example, in a previous study, twenty-four ion-channel genes were found to be differentially expressed between ER-negative and ER-positive tumors [38]. However, in our analysis, we only identified 1 gene, AREG, from the ion-channel family to be predictive of histologic grades of cancer. Thus, many of the 430 breast-cancer related genes may not be predictive of histologic grades but are expected to be highly correlated with the mandatory covariates, i.e. ER and PgR statuses. As suggested by both theoretical and simulation studies, the ridle can be advantageous when mandatory variables are correlated with the irrelevant optional ones. Results from the gene expression analysis further validate and demonstrate the performances of the ridle under this commonly seen scenario.

## Discussion

In this article, we proposed the ridle for sparse regression with mandatory covariates. We provided both theoretical and simulation studies that demonstrated the efficacy of our method. In particular, our results suggest that the ridle may outperform the lasso and elastic net when mandatory covariates are correlated with the irrelevant optional predictors or are highly correlated among themselves. The ridle can also improve upon performances of the lasso and elastic net when mandatory covariates have small or moderate effects.

We employed the *L*1-norm penalty to induce sparsity on the optional set. This is chosen for its simplicity, computational ease, and successes in a myriad of applications; for example, *L*1-norm penalized regressions have been successfully applied in large-scale genome-wide association [3] and eQTL data studies [49]. However, other sparse regularization methods, such as the SCAD [7], adaptive lasso [8], Dantzig selector [9], etc. can also be utilized in place of the *L*1-norm penalty in (1).

The ridle is related to the elastic net [41] that also employs both the *L*1-norm and *L*2-norm penalties. However, the elastic net applies both penalties upon all coefficients of the optional set, whereas the ridle applies the *L*1-norm to coefficients of the optional set and the *L*2-norm to coefficients of the mandatory set for simultaneous estimation of mandatory covariates while allowing selection for others.

In this article, we applied our method in an interesting application to gene expression analysis where we identified more genes related to tumor grade while incorporating clinicopathological variables as mandatory covariates. In addition, the ridle can be applied in a myriad of other genomic studies where mandatory covariates are routinely required, such as when clinical, demographical, or experimental effects have to be incorporated in regression analysis of genomic data sets.

## Conclusions

In this article, we proposed the ridle as a principled sparse regression method for the selection of optional variables while incorporating mandatory ones. Mandatory covariates are routinely encountered in the analysis of genetic-biomedical data. For example, additional covariates describing clinical, demographical or experimental effects need to be included a priori without subjecting them to variable selection. Results suggest that the ridle may outperform current methods when mandatory covariates are correlated with the irrelevant optional predictors or are highly correlated among themselves.

## Additional file

Additional file 1 Proof of theoretical results. This file includes the proofs of Theorems 1–3 in “Methods” section. (210 KB PDF)

## Abbreviations

NPI: Nottingham Prognostic Index
OLS: ordinary least squares
Ridle: Ridge-lasso hybrid estimator
SCAD: smoothly clipped absolute deviation
