# Supplementary material for: Ridle for sparse regression with mandatory covariates with application to the genetic assessment of histologic grades of breast cancer
Source: BMC Med Res Methodol. 2017 Jan 25;17:12. doi: 10.1186/s12874-017-0291-y (PMC5267467; doi:10.1186/s12874-017-0291-y)
Supplement: Additional file 1 — Proof of theoretical results. This file includes the proofs of Theorems 1–3 in “Methods” section. (210 KB PDF) [file 12874_2017_291_MOESM1_ESM.pdf]

RESEARCH

Additional file 1 — Proof of theoretical results in "Ridle for Sparse Regression with Mandatory Covariates with Application to the Genetic Assessment of Histologic Grades of Breast Cancer"

Jing Zhai[1], Chiu-Hsieh Hsu[1] and Z. John Daye[2*]

[*]Correspondence: zhongyindaye@gmail.com
[2]Epidemiology and Biostatistics Department, University of Arizona, USA
Full list of author information is available at the end of the article

Proof of Theorem 1

Let

$$\Phi_n(\mathbf{u}) = \|\mathbf{y} - \mathbf{X}(\beta^0 + \frac{\mathbf{u}}{\sqrt{n}})\|^2 + \lambda_1 \sum_{j \in \mathcal{O}} |\beta_j^0 + \frac{u_j}{\sqrt{n}}| + \lambda_2 \|\beta_{(3)}^0 + \frac{\mathbf{u}_{(3)}}{\sqrt{n}}\|^2.$$

Setting $\hat{\mathbf{u}}^{(n)} = \arg\min \Phi_n(\mathbf{u})$ and $\mathbf{W}^n = (\mathbf{X}^n)^T \epsilon^n / \sqrt{n}$. Then,

$$\begin{aligned} V^{(n)}(\mathbf{u}) &= \Phi_n(\mathbf{u}) - \Phi_n(\mathbf{0}) \\ &= \mathbf{u}^T \mathbf{C}^n \mathbf{u} - 2(\mathbf{W}^n)^T \mathbf{u} + 2\tfrac{\lambda_2}{\sqrt{n}} \mathbf{u}_{(3)}^T \beta_{(3)}^0 + \tfrac{\lambda_2}{n} \mathbf{u}_{(3)}^T \mathbf{u}_{(3)} \\ &\quad + \tfrac{\lambda_1}{\sqrt{n}} \textstyle\sum_{j \in \mathcal{O}} \sqrt{n}(|\beta_j^0 + \tfrac{u_j}{\sqrt{n}}| - |\beta_j^0|). \end{aligned}$$

With $\lambda_1/\sqrt{n} \to c_1$ and $\lambda_2/\sqrt{n} \to c_2$, Theorem 1 follows by noting that $V^{(n)}(\mathbf{u})$ has a unique minimum and using results from [1] and [2].

Proof of Theorem 2

Let $\hat{\mathbf{u}}^n = \hat{\beta}^n(\lambda_1, \lambda_2) - \beta^0$ and $\mathbf{W}^n = (\mathbf{X}^n)^T \epsilon^n / \sqrt{n}$. Then,

$$|\hat{\mathbf{u}}_{(1)}^n| < |\beta_{(1)}^0| \tag{16}$$

and

$$\hat{\mathbf{u}}_{(2)}^n = 0. \tag{17}$$

imply that $\hat{\beta}^n(\lambda_1, \lambda_2)$ is sign consistent. Further, (17) and KKT conditions imply

$$\mathbf{C}_{11}^n \hat{\mathbf{u}}_{(1)}^n + \mathbf{C}_{13}^n \hat{\mathbf{u}}_{(3)}^n - \frac{\mathbf{W}_{(1)}^n}{\sqrt{n}} = -\frac{\lambda_1}{2n} \text{sign}(\beta_{(1)}^0) \tag{18}$$

$$\mathbf{C}_{31}^n \hat{\mathbf{u}}_{(1)}^n + \mathbf{C}_{33}^n \hat{\mathbf{u}}_{(3)}^n + \frac{\lambda_2}{n} \hat{\mathbf{u}}_{(3)}^n - \frac{\mathbf{W}_{(3)}^n}{\sqrt{n}} = -\frac{\lambda_2}{n} \beta_{(3)}^0 \tag{19}$$

$$|\mathbf{C}_{21}^n \hat{\mathbf{u}}_{(1)}^n + \mathbf{C}_{23}^n \hat{\mathbf{u}}_{(3)}^n - \frac{\mathbf{W}_{(2)}^n}{\sqrt{n}}| \leq \frac{\lambda_1}{2n} \mathbf{1}. \tag{20}$$

Substituting $\hat{\mathbf{u}}_{(3)}^n$ from (19) into (18) gives the following expression for $\hat{\mathbf{u}}_{(1)}^n$

$$\begin{aligned} \hat{\mathbf{u}}_{(1)}^n = &\ (\mathbf{C}_{11}^n - \mathbf{C}_{13}^n (\tilde{\mathbf{C}}_{33}^n)^{-1} \mathbf{C}_{31}^n)^{-1} \\ &\cdot [\tfrac{\mathbf{W}_{(1)}^n}{\sqrt{n}} - \tfrac{\lambda_1}{2n} \text{sign}(\beta_{(1)}^0) - \mathbf{C}_{13}^n (\tilde{\mathbf{C}}_{33}^n)^{-1} (\tfrac{\mathbf{W}_{(3)}^n}{\sqrt{n}} - \tfrac{\lambda_2}{n} \beta_{(3)}^0)]. \end{aligned}$$

Replace $\hat{\mathbf{u}}^n_{(1)}$ in (16) and (20) by the above, then the existence of $\hat{\mathbf{u}}^n$ satisfying (16)-(20) is implied by the following

$$\begin{aligned} |\mathbf{Z}^n| &\leq \sqrt{n}|\beta^n_{(1)}| - \frac{\lambda_1}{2\sqrt{n}}|(\mathbf{C}^n_{11} - \mathbf{C}^n_{13}(\tilde{\mathbf{C}}^n_{33})^{-1}\mathbf{C}^n_{31})^{-1} \\ &\quad \cdot [\frac{2\lambda_2}{\lambda_1}\mathbf{C}^n_{13}\tilde{\mathbf{C}}^n_{33}\beta^0_{(3)} - \text{sign}(\beta^0_{(1)})]| \qquad (21) \\ |\zeta^n| &\leq \frac{\lambda_1}{2\sqrt{n}}(\mathbf{1} - |\mathbf{D}^n\text{sign}(\beta^0_{(1)}) \\ &\quad -\frac{2\lambda_2}{\lambda_1}(\mathbf{D}^n\mathbf{C}^n_{13} - \mathbf{C}^n_{23})(\tilde{\mathbf{C}}^n_{33})^{-1}\beta^0_{(3)}|) \qquad (22) \end{aligned}$$

where $\mathbf{Z}^n = (\mathbf{C}^n_{11} - \mathbf{C}^n_{13}(\tilde{\mathbf{C}}^n_{33})^{-1}\mathbf{C}^n_{31})^{-1}\ (\mathbf{W}^n_{(1)} - \mathbf{C}^n_{13}(\tilde{\mathbf{C}}^n_{33})^{-1}\mathbf{W}^n_{(3)})$ and $\zeta^n = \mathbf{D}^n\mathbf{W}^n_{(1)} - \mathbf{W}^n_{(2)} + (\mathbf{C}^n_{(23)} - \mathbf{D}^n\mathbf{C}^n_{13})\tilde{\mathbf{C}}^n_{33}\mathbf{W}^n_{(3)}$. Now, by (5)-(7) and $\lambda_2/n \to 0$, we have

$$\begin{aligned} \mathbf{Z}^n &\to_d N(0, \sigma^2(\mathbf{C}^n_{11} - \mathbf{C}^n_{13}(\mathbf{C}^n_{33})^{-1}\mathbf{C}^n_{31})^{-1}) \\ \zeta^n &\to_d N(0, \sigma^2(\mathbf{C}^n_{22} - \mathbf{C}^n_{23}(\mathbf{C}^n_{33})^{-1}\mathbf{C}^n_{32} \\ &\quad -\mathbf{D}^n(\mathbf{C}^n_{12} - \mathbf{C}^n_{13}(\mathbf{C}^n_{33})^{-1}\mathbf{C}^n_{32}))), \end{aligned}$$

where $\mathbf{Z}^n$ and ζ^n converge to multivariate normal random variables with mean $\mathbf{0}$ and finite variances. Let $s > 0$ be a constant such that $E(\mathbf{Z}^n_j)^2 \leq s^2$ and $E(\zeta^n_j)^2 \leq s^2$. By condition (10), the probability of either of the events (21) and (22) not being satisfied is bounded above by

$$\sum_{\{j:\, \beta^0_j \in \beta^0_{(1)}\}} P(|\mathbf{Z}^n_j| \geq \sqrt{n}(|\beta^0_j| - \frac{\lambda_1}{2n}|\mathbf{b}^n_j|)) + \sum_{\{j:\, \beta^0_j \in \beta^0_{(2)}\}} P(|\zeta^n_j| > \frac{\lambda_1}{2\sqrt{n}}\eta_j),$$

where $\mathbf{b}^n = (\mathbf{C}^n_{11} - \mathbf{C}^n_{13}(\tilde{\mathbf{C}}^n_{33})^{-1}\mathbf{C}^n_{31})^{-1}[(2\lambda_2/\lambda_1)\mathbf{C}^n_{13}\tilde{\mathbf{C}}^n_{33}\beta^0_{(3)} - \text{sign}(\beta^0_{(1)})]$. By $\lambda_1/n \to 0$, $\lambda_1/\sqrt{n} \to \infty$, and $\lambda_2/\lambda_1 \to c < \infty$,

$$\begin{aligned} &\textstyle\sum_{\{j:\, \beta^0_j \in \beta^0_{(1)}\}} P(|\mathbf{Z}^n_j| \geq \sqrt{n}(|\beta^0_j| - \frac{\lambda_1}{2n}|\mathbf{b}^n_j|)) \\ \leq\ & 2(1 + o(1)) \textstyle\sum_{\{j:\, \beta^0_j \in \beta^0_{(1)}\}} (1 - P(\mathbf{Z}^n_j < \sqrt{n}(|\beta^0_j| - \frac{\lambda_1}{2n}|\mathbf{b}^n_j|))) \\ \leq\ & 2(1 + o(1)) \textstyle\sum_{\{j:\, \beta^0_j \in \beta^0_{(1)}\}} (1 - \Phi(\frac{1+o(1)}{s}\sqrt{n}(|\beta^0_j|))) \to 0, \end{aligned}$$

and

$$\begin{aligned} &\textstyle\sum_{\{j:\, \beta^0_j \in \beta^0_{(2)}\}} P(|\zeta^n_j| > \frac{\lambda_1}{2\sqrt{n}}\eta_j) \\ \leq\ & 2(1 + o(1)) \textstyle\sum_{\{j:\, \beta^0_j \in \beta^0_{(2)}\}} (1 - \Phi(\frac{\lambda_1}{2s\sqrt{n}}\eta_j)) \to 0. \end{aligned}$$

Thus, Theorem 2 follows immediately.

Proof of Theorem 3

If $\hat{\beta}(\lambda_1, \lambda_2)$ is sign consistent, then, by KKT conditions,

$$\mathbf{C}^n_{11}\hat{\mathbf{u}}^n_{(1)} + \mathbf{C}^n_{13}\hat{\mathbf{u}}^n_{(3)} - \frac{\mathbf{W}^n_{(1)}}{\sqrt{n}} = -\frac{\lambda_1}{2n}\text{sign}(\beta^0_{(1)}) \qquad (23)$$

$$\mathbf{C}^n_{31}\hat{\mathbf{u}}^n_{(1)} + \mathbf{C}^n_{33}\hat{\mathbf{u}}^n_{(3)} + \frac{\lambda_2}{n}\hat{\mathbf{u}}^n_{(3)} - \frac{\mathbf{W}^n_{(3)}}{\sqrt{n}} = -\frac{\lambda_2}{n}\beta^0_{(3)} \qquad (24)$$

$$|\mathbf{C}^n_{21}\hat{\mathbf{u}}^n_{(1)} + \mathbf{C}^n_{23}\hat{\mathbf{u}}^n_{(3)} - \frac{\mathbf{W}^n_{(2)}}{\sqrt{n}}| \leq \frac{\lambda_1}{2n}\mathbf{1} \qquad (25)$$

must hold with probability 1. Substituting $\hat{\mathbf{u}}^n_{(1)}$ and $\hat{\mathbf{u}}^n_{(3)}$ from (23) and (24) into (25), we have that

$$\mathbf{D}^n\mathbf{W}^n_{(1)} - \mathbf{W}^n_{(2)} + (\mathbf{C}^n_{(23)} - \mathbf{D}^n\mathbf{C}^n_{13})\tilde{\mathbf{C}}^n_{33}\mathbf{W}^n_{(3)} \geq \frac{\lambda_1}{2\sqrt{n}}(-1+\mathbf{v}) \tag{26}$$

holds with probability 1, where $\mathbf{v} = \mathbf{D}^n\text{sign}(\beta^0_{(1)}) - \frac{2\lambda_2}{\lambda_1}(\mathbf{D}^n\mathbf{C}^n_{13} - \mathbf{C}^n_{23})(\tilde{\mathbf{C}}^n_{33})^{-1}\beta^0_{(3)}$. If condition (11) fails and $\mathbf{v}$ has at least one element greater than 1, then the right hand side of (26) has at least one positive element. However, asymptotic normality $\mathbf{D}^n\mathbf{W}^n_{(1)} - \mathbf{W}^n_{(2)} + (\mathbf{C}^n_{(23)} - \mathbf{D}^n\mathbf{C}^n_{13})\tilde{\mathbf{C}}^n_{33}\mathbf{W}^n_{(3)} \to_d N(0, \sigma^2(\mathbf{C}^n_{11} - \mathbf{C}^n_{13}(\mathbf{C}^n_{33})^{-1}\mathbf{C}^n_{31})^{-1})$ implies that there is a non-vanishing probability that any element on the left hand side of (26) is negative. Theorem 3 follows by contradiction.

Author details
[1]Epidemiology and Biostatistics Department, University of Arizona, 1295 N. Martin, Tucson, AZ 85724 USA.
[2]Epidemiology and Biostatistics Department, University of Arizona, USA.

References
1. Geyer, C.: On the asymptotics of constrained m-estimation. Ann. Stat. **22**, 1993–2010 (1994)
2. Knight, K., Fu, W.J.: Asymptotics for lasso-type estimators. Ann. Stat. **28**, 1356–1378 (2000)
